# V-type H^+^-ATPase in the symbiosome membrane is a conserved mechanism for host control of photosynthesis in anthozoan photosymbioses

**DOI:** 10.1098/rsos.211449

**Published:** 2022-01-26

**Authors:** Katie L. Barott, Angus B. Thies, Martin Tresguerres

**Affiliations:** ^1^ Department of Biology, University of Pennsylvania, Philadelphia, PA 19104, USA; ^2^ Scripps Institution of Oceanography, University of California San Diego, 9500 Gilman Drive, La Jolla, CA 92093, USA

**Keywords:** carbon concentrating mechanism, sea anemone, *Symbiodiniaceae*

## Abstract

In reef-building corals (order Scleractinia) and giant clams (phylum Molluca), V-type H^+^-ATPase (VHA) in host cells is part of a carbon concentrating mechanism (CCM) that regulates photosynthetic rates of their symbiotic algae. Here, we show that VHA plays a similar role in the sea anemone *Anemonia majano*, a member of the order Actinaria and sister group to the Scleractinia, which in contrast to their colonial calcifying coral relatives is a solitary, soft-bodied taxa. Western blotting and immunofluorescence revealed that VHA was abundantly present in the host-derived symbiosome membrane surrounding the photosymbionts. Pharmacological inhibition of VHA activity in individual anemones resulted in an approximately 80% decrease of photosynthetic O_2_ production. These results extend the presence of a host-controlled VHA-dependent CCM to non-calcifying cnidarians of the order Actiniaria, suggesting it is widespread among photosymbiosis between aquatic invertebrates and *Symbiodiniaceae* algae.

## 1. Introduction

Symbiotic associations between animals and microorganisms have facilitated the acquisition of a variety of novel physiological capabilities on relatively short evolutionary time scales [1]. Photosymbiotic associations between marine organisms and dinoflagellates in the family *Symbiodiniaceae* are widespread, providing an excellent opportunity for comparative and evolutionary studies. These associations are often regarded as mutually beneficial, with the algae receiving shelter and nutrients from the animal host in exchange for photosynthetically fixed sugars and other photosynthates [2,3]. However, another view considers these relationships as parasitic, whereby the animal forces the release of photosynthates while simultaneously restricting algal growth and reproduction, two key aspects of algal fitness [4,5]. Elucidating the mechanisms that control photosymbiosis is key for determining their physiological and environmental importance, identifying patterns and ultimately, understanding broader evolutionary principles.

Although all photosymbioses with *Symbiodiniaceae* involve the internalization of the algae into the animal host tissues, the algae can be located either intracellularly or extracellularly, depending on the host species. In cnidarians, algal symbionts reside within a specialized intracellular compartment inside gastrodermal cells termed the symbiosome [6], and these specialized cells are accordingly known as ‘symbiocytes’ [7,8]. By contrast, in giant clams (genus *Tridacna*, Phylum Mollusca), algal symbionts are hosted extracellularly within the stomach lumen, in so-called ‘z-tubules’ that penetrate into the siphonal mantle and are exposed to sunlight [9]. Mounting evidence indicates that internalization of the algae allows the host to actively modify the microenvironment surrounding the symbionts to promote photosynthesis and nutrient exchange. Indeed, the physico-chemical properties in the microenvironment can be quite different from the external environment, and in particular, the symbiosome lumen of corals and hydra is known to be highly acidic [10–13]. However, the underlying molecular mechanisms are just beginning to be explored.

The V-type H^+^-ATPase (VHA) is a membrane-associated proton pump enzyme that takes advantage of the energy from adenosine triphosphate (ATP) hydrolysis to transport H^+^ against its electrochemical gradient and mediates a variety of physiological functions in eukaryotes [14]. In symbiotic corals, VHA is found in the symbiosome membrane and contributes to the acidification of the symbiosome [10]. This acidification is hypothesized to promote the conversion of HCO_3_^−^ and H^+^ into CO_2_, which can then diffuse into the algal cell and reach a high enough concentration to sustain algal photosynthesis. In turn, continuous CO_2_ fixation ensures CO_2_ will preferentially diffuse towards the alga rather than towards the host cell cytoplasm. Indeed, pharmacological VHA inhibition results in decreased photosynthetic rate, but only when the symbiosis is intact [10]. In giant clams, host VHA is present in the apical membrane of the epithelial cells that line the z-tubules and surround the algal symbionts; similarly to coral, pharmacological VHA inhibition also decreases algal photosynthesis in giant clam [15]. Taken together, these data suggest that VHA is part of an evolutionarily conserved host-controlled carbon concentrating mechanism (CCM) that can promote photosynthesis in aquatic photosymbioses.

*Anemonia majano* is a solitary sea anemone that belongs to the same subclass as reef-building corals, *Hexacorallia*, and also establishes a photosymbiotic association with *Symbiodiniaceae* alga. However, *A. majanio* and reef-building corals belong to distantly related orders, Actiniaria and Scleractinia, respectively. Furthermore, unlike reef-building corals and giant clams, *A. majano* lacks an external calcium carbonate skeleton. Here, we used similar approaches to those used in coral [10] and giant clam [15] to investigate whether VHA from anemone host cells promotes algae photosynthesis in *A. majano*. Specifically, we used immunohistochemistry to investigate whether VHA is present in the symbiosome membrane of gastrodermal cells and O_2_ measurements in the presence of a highly specific VHA inhibitor [16] to examine whether VHA activity promotes endosymbiotic algal photosynthesis in this non-calcifying invertebrate. This work adds to our physiological and evolutionary understanding of the mechanisms that sustain animal–algal photosymbioses.

## 2. Methods

### 2.1. Sea anemone cultures

Cultures of the symbiotic sea anemone *A. majano* were obtained from the Birch Aquarium at Scripps and kept in a flow-through seawater system at 26°C on a 12 : 12 h light–dark cycle at the Scripps Institution of Oceanography.

### 2.2. Western blotting

Anemones were homogenized in S22 buffer (450 mM NaCl, 10 mM KCl, 58 mM MgCl_2_, 10 mM CaCl_2_, 100 mM Hepes, pH 7.8) supplemented with protease inhibitor cocktail (Sigma) and phosphatase inhibitors (PhosStop; Roche Applied Science, Penzberg, Germany) using a mortar and pestle. This crude homogenate was subjected to slow-speed centrifugation (3000×g for 5 min at 4°C) to enrich for the epidermis (supernatant) and gastrodermis (pellet). The pellet was resuspended in the homogenization buffer. The epidermis and gastrodermis fractions were then subjected to high-speed centrifugation (23 000×g for 30 min at 4°C) into a soluble fraction (supernatant) and a membrane-enriched fraction (pellet). Protein concentrations in all fractions were measured using a Bradford Assay with a bovine serum albumin standard curve (BioRad, Hercules, CA, USA). Equal amounts of protein (3 µg) from each fraction were then incubated in Laemmli Sample buffer with 5% (v/v) β-mercaptoethanol for 15 min at 70°C and loaded on an sodium dodecyl sulphate–polyacrylamide gel electrophoresis gel. Proteins were initially separated at 60 V for 15 min, followed by 200 V until proteins were sufficiently separated. Transfer onto a polyvinylidene difluoride (PVDF) membrane was performed at 25 V for 30 min using a semi-dry apparatus (TurboBlot, BioRad). The PVDF membrane was incubated for 1 h on an orbital shaker in blocking buffer (Tris-buffered saline with 0.1% Triton-X (TBS-T) and 5% fat-free milk) at room temperature and then incubated overnight at 4°C with 1.5 µg ml^−1^ polyclonal anti-VHA_B_ antibodies in blocking buffer. These antibodies recognize the epitope AREEVPGRRGFPGY, which is 100% conserved in animals ranging from cnidarians to human [10], also see [15,17–21]. The PVDF membrane was then washed three times in TBS-T for 10 min each and incubated with secondary antibodies (goat anti-rabbit HRP) for 1 h at room temperature with shaking. The PVDF membrane was washed again, and bands were visualized using the ECL Prime Western Blot Detection Reagent (GE Healthcare, Chicago, IL, USA) and imaged with a Chemidoc Imager (BioRad).

### 2.3. Immunohistochemistry

Anemones were relaxed in 3.5% MgCl_2_ in seawater for approximately 1 min and fixed in 3% paraformaldehyde overnight at 4°C. Tissues were dehydrated, embedded in paraffin and sectioned followed previously described protocols [22]. Briefly, tissue sections were rehydrated, permeabilized in 0.2% triton-x in phosphate buffered saline (PBS) and incubated in blocking buffer (2% normal goat serum and 0.5% keyhole limpet haemocyanin in PBS) for 60 min at room temperature and then incubated with 3 µg ml^−1^ anti-VHA_B_ antibodies in blocking buffer overnight at 4°C. Sections were rinsed in cold PBS-T and incubated with 4 µg ml^−1^ secondary antibodies (goat anti-rabbit IGG Alexa-555). Nuclei were stained with 1 µg ml^−1^ Hoescht for 5 min. Tissue sections were then visualized using an epifluorescence microscope with structured illumination (Zeiss AxioObserver Z1 with Apotome 2, Carl Zeiss AG, Oberkochen, Germany) connected to a metal halide lamp (HXP 120C Compact Light Source, Leistungselektronik, Stockholmer, Germany). Alexa 555 signal was visualized using filter set 43 He (Ex. 550/25 nm, Beamsplitter 570 nm, Em. 605/70 nm), DAPI was visualized using filter set 49 He (Ex. 365 nm, Beamsplitter 395 nm, Em. 445/50 nm) and algal chlorophyll was visualized using filter set 38 He (Ex. 470/40 nm, Beamsplitter 495 nm, Em. 525/50 nm). Digital images were adjusted for brightness and contrast using Zeiss Axiovision software.

### 2.4. Oxygen production rates

Individual anemones were placed in 5 ml sealed glass chambers filled with 0.2 µm filtered seawater (FSW). Chambers were kept at 26°C by immersing the vials in a water bath, and the water within each chamber was circulated using a magnetic stir bar. Anemones were illuminated with 150 µmol m^−2^ s^−1^ photosynthetically photon flux density. A Clark-type oxygen electrode (Unisense, Aarhus, Denmark) was inserted into the vial through a capillary port in the lid, and the dissolved oxygen (DO) concentration was recorded once per second (MicOx software; Unisense). The oxygen probe was calibrated prior to each use with a two-point calibration curve using a freshly prepared solution of 0.1 M NaOH, 0.1 M sodium ascorbate (0% DO) or 100% air-saturated seawater. Oxygen evolution rates for each anemone were measured in FSW until a steady slope was attained for at least 15 min. The water was then replaced with FSW with either 0.5% v/v DMSO (carrier control) or 500 nM bafiloymycin A1 (Sigma, St. Louis MO, USA) dissolved in 0.5% v/v DMSO, and oxygen evolution rates were measured for 30 min. A paired *t*-test was used to test for significant differences in oxygen evolution in each anemone between the FSW incubation versus DMSO or bafiloymycin A1 incubation (*n* = 5 per treatment).

## 3. Results

**Figure 1.**
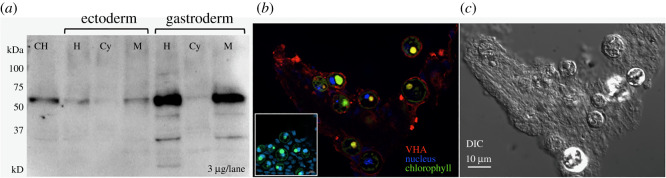
VHA protein expression and localization in *Anemonia majano*. (*a*) Western blot showing VHA protein expression in *A. majano* tissues. Crude tissue homogenates (CH) were separated into ectodermis and gastrodermis fractions (H) by differential centrifugation, and then each of those fractions were further fractionated into the cytosolic (Cy) and membrane fractions (M). 3 µg of protein from each fraction was loaded in each lane. (*b*) Immonohistochemical detection of VHA in *A. majoano* gastroderm. Red = VHA immunofluorescence signal; blue = DAPI nuclear staining; green = chlorophyll autofluorescence. Inset: peptide preabsorption control showing no signal. (*c*) Differential interference contrast (DIC) image.

**Figure 2.**
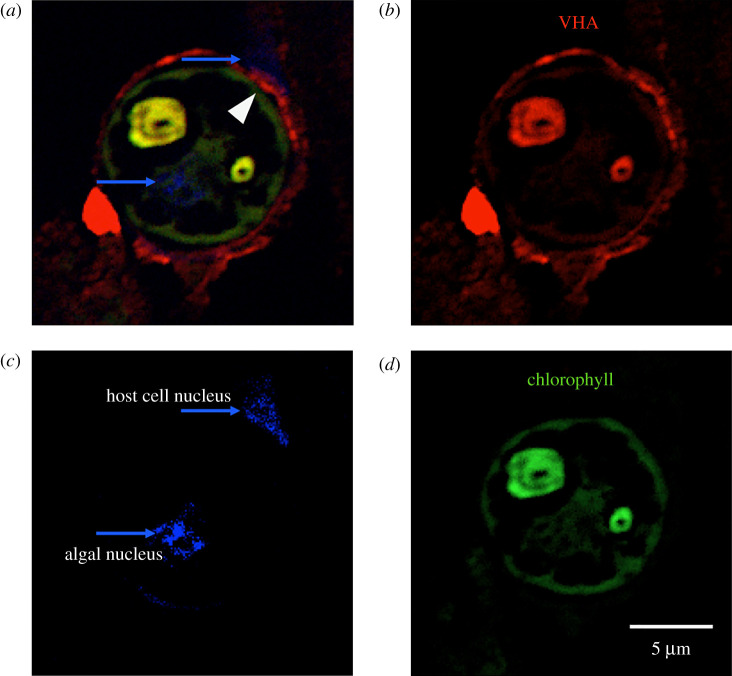
VHA is present in the symbiosome membrane of *A. majano* algal-containing gastrodermal cells. (*a*) Composite image showing VHA immunofluorescence signal (red), DAPI nuclear staining (blue) and chlorophyll autofluorescence (green). (*b*) VHA, (*c*) DAPI and (*d*) chlorophyll individual channels. Notice that VHA is present in the thin region between the host cell nucleus (blue arrow in *c*) and the algae, indicative of symbiosomal localization (white arrowhead in *a*).

Western blotting using specific anti-VHA_B_ antibodies showed the expected approximately 55 kDa band in both ectodermal and gastrodermal anemone tissues (figure 1*a*). Expression was highest in the gastrodermis, where the algal symbionts are located (figure 1*a*). Within the gastrodermis, VHA was enriched in the membrane fraction that includes the symbiosome membranes. Indeed, immunohistochemistry confirmed the localization of VHA in gastrodermal cells (figure 1*b,c*). Higher magnification observations revealed that VHA was specifically present in the symbiosome membrane surrounding the algal cells (figure 2), as evidenced by the expression of VHA between the algal perimeter and the host cell nucleus (figure 2*c*).

Finally, we examined the effects of the highly specific VHA inhibitor, bafilomycin A1, on O_2_ evolution rate as a proxy for photosynthetic activity. Control anemones immersed in FSW produced approximately 300 nmol O_2_ h^−1^, and this rate was not significantly affected upon exposure to FSW spiked with DMSO, the vehicle solvent for bafilomycin A1. On the other hand, treatment with 500 nM bafilomycin A1 resulted in a significant approximately 80% decrease in O_2_ evolution down to approximately 80 nmol O_2_ h^−1^ (table 1).

**Table 1.** Effect of VHA inhibition on O_2_ production rate by *A. majano*. Individual anemones were placed in a respirometry chamber under 150 μmol m^−2^ s^−1^ light, in the presence of solvent *DMSO* or 500 nM bafilomycin A1. Superscripts indicate significant differences (*N* = 5). ‘FSW’ versus ‘Treatment’ column: paired *t-*test; *p < 0.01. ‘Relative to FSW’ column: one sample *t*-test difference from 1, *p* < 0.01.

	FSW	treatment	relative to FSW	*p*-value
DMSO (0.5%)	298.9 ± 14.7	314.8 ± 73.9	1.04 ± 0.2	0.8252
bafilomycin A1 (500 nM)	334.9 ± 41.4	79.9 ± 63.0*	0.2 ± 0.16*	0.0047
	nmol O_2_ h^−1^			

## 4. Discussion

Our results indicate that *A. majano* uses VHA located in the symbiosome membrane of symbiocytes to promote photosynthesis by their endosymbiotic *Symbiodiniaceae* algae. This mechanism was originally described in Scleractinian corals, where VHA activity was shown to acidify the symbiosome lumen down to pH approximately 4 and to promote photosynthetic O_2_ production [10]. An analogous mechanism has since been reported in the photosymbiotic association between giant clams and *Symbiodiniaceae* algae; however, in this case, the VHA is located in the lumen-facing membrane of the epithelial cells that line the z-tubules where the algal symbionts are hosted extracellularly [15]. Thus, the VHA-dependent host-controlled CCM is functional in photosymbiotic associations that differ from each other in some key evolutionary, morphological and physiological aspects, namely, two distantly related invertebrate phyla (Cnidaria and Mollusca), intracellular versus extracellular photosymbiont hosting (coral and anemone versus giant clam, respectively) and calcifying versus non-calcifying organisms (coral and giant clam versus anemone, respectively).

The VHA plays a ubiquitous and essential role in lysosomal and phagosomal function in eukaryotes [23–25], and thus exploring the putative role of VHA in host-controlled CCM in other photosymbiosis cannot be done by datamining genomes and transcriptomes. Instead, this task requires determining VHA's cellular and subcellular localization in the organism of interest, ideally coupled to functional assays. Interestingly, VHA is also abundantly expressed in the chemoautotrophic bacteria-bearing trophosome tissue of *Riftia* hydrothermal vent worms, where it has been proposed to mediate acid–base regulatory processes [26,27]. Furthermore, immunolabelling observations in *Riftia* have indicated that VHA is present in the cells that host the bacteria (termed ‘bacteriocytes’) [28]. Similarly, VHA is abundantly expressed in the ‘root’ tissue of bone-eating *Osedax* worms [18], which also harbours heterotrophic symbiotic bacteria [29,30]. While VHA is most prominently present on the apical membrane of the *Osedax* epithelial cells in contact with the bone substrate, it was also seen in presumed vesicles as well as throughout the trophosome, leaving open a potential role for VHA in symbiosis in this species [18]. However, the observations in both *Riftia* and *Osedax* lack sufficient resolution to determine whether VHA is present in the vacuolar membrane that surrounds the bacteria within *Riftia* and *Osedax* bacteriocytes, which would be analogous to the symbiosome membrane within cnidarian symbiocytes. Additionally, there is no functional evidence for the role in VHA in *Riftia* and *Osedax* symbioses, a challenging task given the deep-sea habitat of these two worms. In any case, the emerging picture is that VHA is an important and evolutionary component of photosynthetic, chemoautotrophic and heterotrophic associations between marine invertebrates and microbes.

Many aspects of this VHA-dependent host-controlled CCM remain unknown and deserve further investigation. For example, dissolved inorganic carbon must be transported across several cellular membranes and delivered to the algal microenvironment, where the acidic pH generated by the VHA would promote its conversion into CO_2_ for use in photosynthesis. While we can presume that this role is fulfilled by bicarbonate transporters, their molecular identity and kinetic properties may vary as a function of phylogenetic, species-specific and environmental aspects. In addition, the VHA-dependent acidification of the microenvironment surrounding photosymbionts could play other important roles such as halting cell division and energizing the transport of nitrogen and other nutrients (discussed in [8,14,31,32]). These mechanisms would also require additional yet unidentified effector proteins that underly differential physiological responses and regulation, and as such, they could explain differential adaptations to specific environments and responses to stressors.

## Data accessibility

Data and relevant code for this research work are stored in GitHub: https://github.com/BarottLab/AmajanoVHA2013 and have been archived within the Zenodo repository: https://doi.org/10.5281/zenodo.5772774.
